# Rilpivirine versus etravirine validity in NNRTI-based treatment failure in Thailand

**DOI:** 10.7448/IAS.17.4.19740

**Published:** 2014-11-02

**Authors:** Phairote Teeranaipong, Sunee Sirivichayakul, Suwanna Mekprasan, Kiat Ruxrungtham, Opass Putcharoen

**Affiliations:** 1 Department of Parasitology, Faculty of Medicine, King Chulalongkorn Memorial Hospital, Chulalongkorn University, Bangkok, Thailand; 2 Division of Allergy and Clinical Immunology, King Chulalongkorn Memorial Hospital, Chulalongkorn University, Bangkok, Thailand; 3 Division of Infectious Diseases, King Chulalongkorn Memorial Hospital, Chulalongkorn University, Bangkok, Thailand

## Abstract

**Introduction:**

Etravirine (ETR) and rilpivirine (RPV) are the second-generation non-nucleoside reverse transcriptase inhibitors (NNRTI) for treatment of HIV-1 infection. Etravirine is recommended for patients with virologic failure from first generation NNRTI-based regimen [1]. RPV has profile with similar properties to ETR but this agent is approved for treatment-naïve patients [2]. In Thailand, ETR is approximately 45 times more expensive than RPV. We aimed to study the patterns of genotypic resistance and possibility of using RPV in patients with virologic failure from two common NNRTI-based regimens: efavirenz (EFV)- or nevirapine (NVP)-based regimen.

**Materials and Methods:**

Data of clinical samples with confirmed virologic failure during 2003–2010 were reviewed. We selected the samples from patients who failed EFV- or NVP-based regimen. Resistance-associated mutations (RAMs) were determined by IAS-USA Drug Resistance Mutations. DUET, Monogram scoring system and Stanford Genotypic Resistance Interpretation were applied to determine the susceptibility of ETR and RPV.

**Results:**

A total of 2086 samples were analyzed. Samples from 1482 patients with virologic failure from NVP-based regimen treatment failure (NVP group) and 604 patients with virologic failure from EFV-based regimen treatment failure (EFV group) were included. 95% of samples were HIV-1 CRF01_AE subtype. Approximately 80% of samples in each group had one to three NNRTI-RAMs and 20% had four to seven NNRTI-RAMs. 181C mutation was the most common NVP-associated RAM (54.3% vs 14.7%, p<0.01). 103N mutation was the most common EFV-associated RAM (56.5% vs 19.1%, p<0.01). The calculated scores from all three scoring systems were concordant. In NVP group, 165 (11.1%) and 161 (10.9%) patients were susceptible to ETR and RPV, respectively (p=0.81). In EFV group, 195 (32.2%) and 191 (31.6%) patients were susceptible to ETR and RPV, respectively (p=0.81). The proportions of viruses that remained susceptible to ETR and RPV in EFV group were significantly higher than NPV group (ETR susceptibility 32.2% vs 11.1%, p<0.01, RPV susceptibility 31.6% vs 10.9%, p<0.01), respectively.

**Conclusions:**

RPV might be a cost saving and reasonable second line NNRTI for patients who failed EFV- or NVP-containing regimens, especially in resource-limited setting because these two agents have comparable susceptibility identified by genotyping. From our study, approximately 30% of patients who failed EFV-based regimens had viruses that remained susceptible to RPV.
